# Work and health during the COVID-19 crisis among Dutch workers and jobseekers with (partial) work disabilities: a mixed methods study

**DOI:** 10.1186/s12889-023-15720-w

**Published:** 2023-05-26

**Authors:** Mara de Visser, Marloes de Graaf-Zijl, Johannes R. Anema, Maaike A. Huysmans

**Affiliations:** 1 Amsterdam UMC Location Vrije Universiteit Amsterdam, Public and Occupational Health, Van Der Boechorstraat 7 1081 BT, Amsterdam, the Netherlands; 2 Amsterdam Public Health Research Institute, Societal Participation and Health, Amsterdam, the Netherlands; 3 UWV (Social Security Institute), Kenniscentrum, La Guardiaweg 94-114, 1043 DL Amsterdam, the Netherlands

**Keywords:** COVID-19, Disability, Employment, Working conditions, Perceived health

## Abstract

**Background:**

The consequences of restrictive measures during the COVID-19 outbreak have potentially been enormous, especially for those in a vulnerable position in the labour market. This study aims to describe the impact of the COVID-19 crisis on work status, working conditions and health among people with (partial) work disabilities—with and in search of work—during the COVID-19 pandemic in the Netherlands.

**Methods:**

A mixed methods design was used, combining a cross-sectional online survey and ten semi-structured interviews with people with a (partial) work disability. The quantitative data included responses to job-related questions, self-reported health, and demographics. The qualitative data consisted of participants’ perceptions about work, vocational rehabilitation, and health. We used descriptive statistics to summarize the responses, conducted logistic and linear regression and integrated our qualitative findings with the quantitative findings, aiming at complementarity.

**Results:**

Five hundred and eighty-four participants (response rate 30.2%) completed the online survey. The majority of participants experienced no change in work status: 39 percent remained employed, 45 percent remained unemployed, six percent of respondents lost their job, and ten percent became employed during the COVID-19 crisis. In general, the results showed a deterioration in self-rated health during the COVID-19 outbreak, both for participants at work and in search of work. Participants who lost their job during the COVID-19 crisis reported the highest deterioration in self-rated health. Interview findings revealed that loneliness and social isolation were persistent during the COVID-19 crisis, especially among those in search of work. Additionally, employed participants identified a safe work environment and the possibility to work at the office as important factors for overall health.

**Conclusions:**

The vast majority of study participants (84.2%) experienced no change in work status during the COVID-19 crisis. Nonetheless, people at work and in search of work encountered barriers to maintaining or (re)gaining employment. People with a (partial) work disability who lost their job during the crisis appeared to be most affected in terms of health. Employment and health protections could be strengthened for persons with (partial) work disabilities in order to build resilience in times of crisis.

**Supplementary Information:**

The online version contains supplementary material available at 10.1186/s12889-023-15720-w.

## Background

Having a paid and meaningful job is essential for socio-economic well-being [1, 2]. Being employed improves financial independence, self-esteem, and confidence; facilitates access to healthcare; and contributes to social connectedness and de-stigmatization of mental illness [3–5]. This is especially true for people with work disabilities (WD). WD refers to the (partial) inability to engage in gainful employment due to physical or mental illness [6]. The relationship between work and health has been well documented across a wide range of disability groups [7–9]. Poor working conditions and poor job satisfaction can negatively affect health, and in turn declining health undermines employability [10]. Despite the introduction of a variety of measures to improve employment among disability groups in the Netherlands—such as financial incentives for employers hiring persons with disabilities— individuals with disabilities continue to face challenges when it comes to job participation [7, 11, 12].

Since March 2020, the COVID-19 crisis has had a radical impact on society. The effects of this period on the labour market and income are a result of not only the COVID-19 crisis itself but also of policy responses to the crisis. Dutch measures to control the spread of the virus were comprised of restrictive measures, such as people keeping a 1.5-m distance from one another, wearing face masks in public areas, and working from home. Additionally, the Dutch government responded with a diverse set of economic support instruments such as wage subsidies and loan guarantees to help companies avoid bankruptcy and to support workers in staying employed.

Globally, only a few studies have been conducted to examine the impact of the pandemic on employment status among vulnerable populations. COVID-19-related job loss disproportionally affected people with lower levels of education, women, and the elderly [8, 9]. In terms of public health consequences, several studies suggest that working conditions deteriorated during the crisis and that employees were more likely to have mental health problems during the COVID-19 crisis [13, 14]. Internationally, becoming unemployed during the COVID-19 pandemic has been correlated with detrimental health outcomes, such as psychological distress, anxiety, and depressive symptoms [14–16]. Social isolation, uncertainty, and conflicting messages from authorities have been described as some of the main factors negatively affecting mental health and well-being [17, 18]. The impact of the COVID-19 crisis on health, particularly among vulnerable groups, is a global concern because it could widen pre-existing health inequalities. Yet, little is known about the impact of the COVID-19 crisis on employment, working conditions, and perceived health among vulnerable individuals. Investigation of the overall impact of the COVID-19 crisis for individuals with disabilities is needed to understand societal and health-related barriers and to reveal the needs of this vulnerable group in times of crisis.

The present study aims to describe the relationship between the COVID-19 crisis and work status, working conditions, and health among people with (partial) WD, applying a mixed methods approach. Additionally, it examines changes in self-rated health (SRH) among persons with (partial) WD before and during the COVID-19 crisis and whether (changes in) work status can be associated with changes in SRH.

## Methods

### Study design

We applied a mixed methods approach to answer the research questions. Our data sources included a cross-sectional, web-based survey which was sent to people with (partial) work disabilities, complemented by ten qualitative semi-structured interviews with survey participants.

### Study population

This study concerned people with (partial) work disabilities (WD). In the Netherlands, people with reduced work capacity due to chronic health conditions are eligible to receive a work disability benefit under the Work and Income (Capacity for Work) Act (WIA), which is provided by the Dutch Social Security Institute (SSI). SSI is an administrative authority in the Netherlands that helps clients to remain in or find employment and that evaluates illness and employment incapacity. During the application for a disability benefit, an insurance physician (IP) employed by the SSI performs a medical assessment. The IP lists disorders according to the Dutch Classification of Occupational Health and Social Insurance (CAS). The CAS is based on the International Statistical Classification of Disease and Related Health Problems (ICD-10). Young disabled people (18–30 years old), who are (partially) incapacitated for work may be eligible to receive a benefit under the Invalidity Insurance (Young Disabled Persons) Act (Wajong).

For the present study, participants were recruited through an online panel (*n* = 1933) provided by the SSI. Inclusion criteria were (1) registered at SSI as WIA (Work and Income according to Labour Capacity Act), WGA (Return to Work (Partially Disabled) Regulations), or Wajong (Disablement Assistance Act for Handicapped Young Persons); (2) receiving a benefit under either the WIA Act or Wajong Act and (3) aged 18 and above.

### Quantitative data collection

**Fig. 1:**
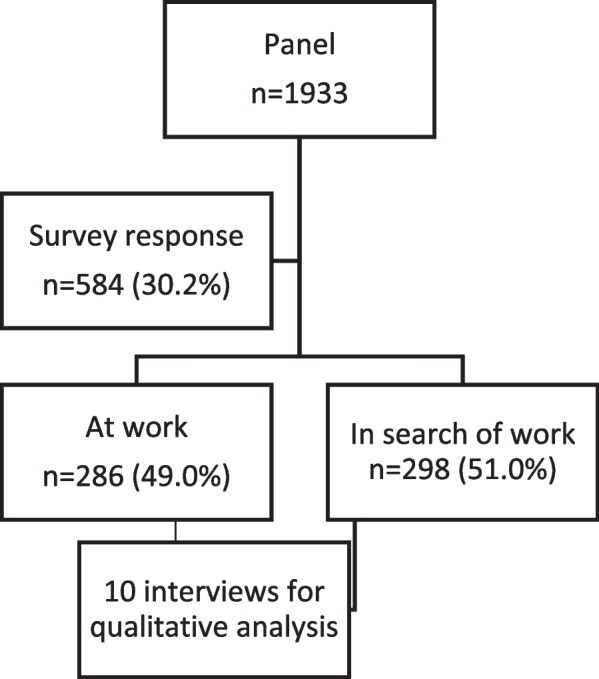
Sample flow diagram

Data collection was carried out between May and June 2021. During this period, the government financially supported employers with benefits for their employees (i.e. supplemented wages) to avoid employees being fired. In addition to enforcing a lockdown (from March 2020 to June 2020 and from October 2020 to June 2021), restrictive measures such as keeping 1.5-m distance, wearing a face mask in public areas, and working from home were still advised in the Netherlands. Data was collected through a web-based survey of panel members from SSI. Before use, the survey was tested and checked by co-researchers and employees of SSI. An email was sent to each panel member containing a brief explanation of the study’s objective and privacy conditions related to participation, along with a link to the survey. Participants were informed that by completing the survey they would give their consent for the data to be used for research purposes. Participation was voluntary and participant anonymity and confidentiality were assured and emphasized. The surveyconsisted of 31 items, both open-ended and closed-ended questions, and took approximately 15 min to complete. The final sample consisted of 584 respondents, whereby groups ‘At work’ (49.0%) and ‘In search of work’ (51.0%) were equally sized. Figure 1 is a flow diagram illustrating how the sample was achieved.

### Survey measures

Participants were asked to provide information about their sex, age, household composition, educational level, and type of work contract. From there, two different questionnaires were used depending on the participant’s employment status (employed or unemployed). Employed participants were asked about changes in work, work functioning, and job satisfaction during the COVID-19 crisis. Unemployed participants were asked about their perspectives on job opportunities and willingness to work during the COVID-19 crisis. For participants receiving vocational rehabilitation (VR) (i.e., assistance to prepare for, secure, retain, or regain employment), additional questions were asked about changes in VR and their satisfaction with the coaching they received during the COVID-19 pandemic. SRH measurements were collected to understand participants’ perceptions of their own health, subdivided into physical health, mental health, and financial distress.

#### Perceived impact of COVID-19 on (finding) work

The impact of the COVID-19 outbreak was assessed among employed people according to four points specifically designed for this survey: (1) work more/less hours a week; (2) work takes more/less energy; (3) wearing a face mask at work/work from home/instructed to do other tasks/ other; (4) job satisfaction on a five-point Likert scale (1 = very satisfied, 2 = satisfied, 3 = neutral, 4 = dissatisfied, 5 = very dissatisfied).

Among unemployed persons, the following items examined the extent to which the COVID-19 outbreak influenced perceived job opportunities: (1) perceived chance to find a paid job within six months ( 1 = very likely, 2 = likely, 3 = neutral, 4 = unlikely, 5 = very unlikely); (2) worrying about finding a paid job (1 = not at all, 2 = not really, 3 = neutral, 4 = somewhat, 5 = very much); and (3) willingness to find a paid job (1 = very low, 2 = low, 3 = neutral, 4 = high, 5 = very high).

#### Perceived impact of COVID-19 on health

Health status was assessed according to self-rated health (SRH) (both physical and mental) before and during the COVID-19 outbreak: (1) At this moment, how would you evaluate your physical health?; (2) In general, how would you evaluate your physical health before the COVID-19 outbreak?; (3) At this moment, how would you evaluate your mental health?; (4) In general, how would you evaluate your mental health before the COVID-19 outbreak?

Financial distress, a component of mental health, was investigated separately: (5) At this moment, do you have any worries about your financial situation?; (6) In general, did you have any worries about your financial situation before the COVID-19 outbreak?

Responses to the above questions were measured on a five-point Likert scale: 1 = very good/no worries at all, 2 = good/no worries, 3 = fair/neutral, 4 = poor/some worries, 5 = very poor/worried a lot.

### Qualitative interview participants and recruitment

Interview participants were recruited from our survey. Out of all voluntary registered participants, we selected ten of them aiming for diversity in terms of age, gender and employment status. We informed all participants about the study and privacy regulations both by phone and email. The research team developed interview guides—one for employed and one for unemployed individuals—to further explore survey findings and to assist in interpretations. The interview guides were pilot tested by two researchers and an Expert by Experience co-researcher. Themes were assessed using open-ended questions and follow-up probes and included perceptions of the impact of the COVID-19 outbreak on work, the workplace, and health. Interviews were conducted in October and November 2021. During that time, rising COVID-19 infections led to strict lockdown measures. Interviews were carried out online in Dutch by two researchers and lasted approximately one hour. Prior to the interviews, the researchers gathered informed consent, which was audio-recorded separately. Interview participants received 15 euros as compensation.

### Data analysis

#### Quantitative analysis

Baseline characteristics were presented as summary statistics, wherein frequency estimates and proportions were used for categorical variables. Oneway-Anova and Bonferroni posthoc tests were performed to compare means on self-rated physical and mental health and financial distress before and during the COVID-19 crisis by subgroups according to work status. Dependent variables on perceived health were measured on a five-point Likert scale (1 = very good, 2 = good, 3 = fair, 4 = poor, 5 = very poor) and collapsed into a binary outcome (1 = good, 2 = poor), so that scores between 1 and 3 represented ‘good’ and scores of 4 and 5 represented ‘poor’. In analysis 1, multiple logistic regression was utilized to study the relationship between SRH and work status. Adjusted odds ratios (OR) were subsequently generated using 95% confidence intervals (CI). In analysis 2, multivariate linear regression was applied to examine whether changes in work status were related to changes in SRH. Changes in SRH were derived from individual level changes in self-rated health before and during the COVID-19 crisis. All data was analysed using STATA/SE 14.1.

#### Qualitative analysis

Interviews were audio recorded, transcribed verbatim, and coded iteratively using thematic analysis. Due to privacy regulations, survey data could not be linked to individual interview data. Interview data was managed by MAXQDA. The first three transcripts were analyzed by the first researcher (MV), and the codes were checked and discussed by a second researcher (MH). The remaining seven transcripts were then coded by the first researcher (MV). The final coding schemes were discussed until agreement was reached.

## Results

### Sample characteristics

In total, 584 surveys were completed (response rate = 30.2%). Descriptive characteristics of the survey respondents are shown in Table 1. The majority of respondents (84.2%) experienced no change in work status: they stayed employed or stayed unemployed. In a small minority of respondents work status changed: 6.0 percent of employed respondents became unemployed and 9.8 percent of unemployed respondents became employed during the COVID-19 crisis.

**Table 1:** Descriptive characteristics of survey respondents, stratified by work status

	Total N (%)	Employed N (%)	Unemployed N (%)	Became unemployed N (%)	Became employed N (%)
Total	584 (100)	229 (39.2)	263 (45.0)	35 (6.0)	57 (9.8)
Age (years)					
20–29	42 (7.2)	22 (9.6)	14 (5.3)	-	6 (10.5)
30–39	101 (17.3)	44 (19.2)	36 (13.7)	9 (25.7)	12 (21.1)
40–49	116 (19.9)	56 (24.5)	44 (16.7)	6 (16.1	10 (17.5)
50–59	150 (25.7)	52 (22.7)	73 (27.8)	10 (28.6)	15 (22.7)
60 +	153 (26.2)	51 (22.3)	82 (31.2)	8 (22.9)	12 (21.1)
Unknown	22 (3.8)	4 (1.8)	14 (5.3)	2 (5.7)	2 (3.5)
Female	269 (46.1)	93 (40.6)	130 (49.4)	20 (57.1)	26 (45.6)
Have children	135 (23.1)	57 (24.9)	56 (21.3)	8 (22.9)	14 (24.6)
Live alone	199 (34.1)	65 (28.4)	100 (38.0)	14 (40.0)	20 (35.1)
Education					
Higher education or university	202 (35.9)	80 (34.9)	86 (32.7)	16 (45.7)	27 (47.4)
Practical education or secondary school	241 (42.9)	100 (43.7)	111 (42.2)	16 (45.7)	23 (40.4)
No education or primary school	119 (21.2)	49 (21.4)	66 (25.1)	3 (8.6)	7 (12.3)
Receiving vocational rehabilitation	159 (27.2)	55 (24.0)	69 (26.4)	13 (37.1)	22 (38.6)

In the qualitative part of this study, participants (*n* = 10) ranged in age from 28–62. Six were female, and four were living alone. In terms of work status, seven participants were working (employed or became employed) and three were in search of work (unemployed or became unemployed).

### In search of work, maintaining work, and work conditions during the COVID-19 crisis

#### Changes in work conditions

About one third of employed participants (34.1%) experienced an increase in workload during the COVID-19 crisis. Findings also indicate that 13.1 percent of employed participants experienced reduced working hours. Furthermore, a minority of respondents (15.7%) was instructed to take on tasks outside their usual duties during the COVID-19 crisis. About a quarter of the employed participants (29.3%) worked (partly) from home, and a quarter had to wear a face mask at work (27.5%).

#### Perceptions towards work during the COVID-19 crisis

Most participants who were working enjoyed their jobs (61.2%) and felt supported (59.7%). Only 15.2 percent of those who were unemployed before the start of the COVID-19 outbreak believed they would find work, whereas 57.1 percent who lost their job during the crisis believed they would find work.

Furthermore, willingness to work was relatively low among participants in search of work. 31.2 percent of participants who were unemployed before the crisis reported that they were highly willing to work, compared to 57.2 percent of those who became unemployed during the COVID-19 crisis.

#### Vocational rehabilitation during the COVID-19 crisis

In total, 27.7 percent (*n* = 159) of participants received VR during the COVID-19 crisis (employed = 24.0%, became employed = 38.6%, unemployed = 26.2%, became unemployed = 37.1%). In total, 9.4 percent of participants reported that they received more VR during the COVID-19 crisis and 20.1 percent reported that they received less VR than usual. In some cases (21.4%), participants received (partly) remote VR due to the COVID-19 pandemic. Overall, satisfaction with the VR was high. A total of 64.2 percent were satisfied or highly satisfied with the VR they received at the moment the survey was administered. However, unemployed participants were notably less satisfied with their VR (employed = 70.9%, became employed = 68.2%, unemployed = 58.0%, became unemployed = 61.5%).

### Experiences and perspectives related to work

In the qualitative part of the study, participants were asked about their experiences related to work during the COVID-19 crisis and changes in work due to the COVID-19 crisis. Themes, subthemes, and main codes derived from the main theme ‘Work status’ are described below (see also Additional file 1).

Subthemes derived from ‘In search of work’ included self-stigma, hindering and facilitating factors in VR, and decreased motivation due to lack of structure. Subthemes of ‘At work’ were comprised of self-control, disclosure, and working conditions. These subthemes encompassed both COVID-19-related subjects and structural concerns that were also relevant during the COVID-19 crisis. Overall, an important finding of the qualitative analysis is that most issues for both ‘In search of work’ and ‘At work’ seem to lie in patterns of social interaction (with employer or coach). Disparate and dysfunctional (conflict) patterns were mainly observed among participants in search of work, whereas a cooperative attitude was mainly observed among participants at work. Moreover, the sub-themes highlight the contrast in experiences between participants at work and those in search of work.

#### In search of work: Motivation decreased due to lack of structure and issues with communication

Among most participants, motivation and willingness to search for work and start a new job substantially decreased. More importantly, unemployed participants experienced a lack of structure during the day, which hindered them to activate themselves. For instance, one participant explained that intrinsic motivation to switch from one task to another was difficult because there were no appointments or deadlines going on.

At the moment [i.e., during the COVID-19 crisis], I don’t have anything going on in a day. I have no structure. I don’t have to be somewhere at 9 AM, because I have no appointments. I usually manage to do that [i.e. following a daily routine]. But switching by myself [from one task to another], I don’t have that intrinsic motivation. (Woman, 32 years old).

Every day I had a few things to do, a few hours filling up my day. Just a reason to get out of bed. But, yes, then Corona came, and I lost everything. (Woman, 60 years old)

However, motivational issues were not only caused by lack of structure, but also by the experienced difficulties in communication with potential employers.

No response. They would call me back. Again nothing. Yes, at some point, after the third time, I was fed up and I wouldn’t call anymore. (Woman, 44 years old)

#### At work: Disclosure of disabilities and facilitation by employers

Employees who disclosed their disabilities and work-related needs felt understood and accommodated by their employers. Having a safe workplace (i.e., the possibility to maintain COVID-19 preventive measures) and being allowed to work at the office were mentioned as much needed.

After three days of working from home I requested to work at the office, which was granted. (Man, 31 years old)

We need to keep distance. If that’s not possible, it’s a face mask. But I have indicated, if there is too much hustle and bustle, then I will go out and do something else, pick up cups or something. (Woman, 33 years old).

#### At work: working conditions

The importance of a workplace outside the house came up in most interviews among office workers. Although the benefits of working from home were acknowledged, the associated challenges were also strongly emphasized. Participants mentioned that they needed separation between work and their private life.

I need an environment where I know I need to do this and that now. At home, I will do too many things that are not related to work or study. And then in the end you just do too little or nothing. (Man, 31 years old).

Additionally, workers explained that being connected with colleagues was essential for them. Being known and staying in face-to-face contact with colleagues ensured that workers received understanding and compassion. In addition, online work meetings often caused misunderstandings and uncertainty in communication.

So, yes, for me it’s very important that my colleagues know who I really am. Because, yes, they should know I’m not that kind of person to be sick for a day. (Woman, 29 years old)

Then you will call each other again, after that (online) meeting. Yes, did I really understand you, or what do you mean by that? (Man, 31 years old)

On the other hand, some participants at work experienced anxiety and frustration because their workplace felt unsafe (i.e., not being able to maintain preventive COVID-19 measures).

#### In search of work: Hindering and facilitating factors in vocational rehabilitation

Participants in search of work mentioned both hindering and facilitating factors regarding VR. These factors were not only relevant during the COVID-19 crisis but were prevalent before the pandemic. Hindering factors included feeling unheard and lacking an experience of autonomy. Feeling connected with the VR coach (i.e., feeling appreciated and heard) was mentioned as a facilitating factor in VR. Facilitation in job perspectives that were suitable and satisfying was mentioned as another important element. Experiences of receiving online VR were generally reported to be positive or neutral.

We kept in contact throughout the corona crisis. And we still keep in touch. And I just hope I can do something, even if it is for a few hours and that I like the job. If I did something that did not suit me, yes, then it would not work for me, then I would be home again within a month or two months. (Woman, 44 years old).

I receive coaching, but she also knows a bit about what I have been through. And she said: ‘You’re doing very well. You do the work well and you have been working here for six months now. You have been holding on for so long now’. (Woman, 32 years old).

#### In search of work: Self-stigma, feelings of uncertainty, and not feeling good enough for the job

Across interview findings, internalized stigma came up frequently as an underlying theme among participants in search of work. Uncertainty about job application procedures and the feeling of not being good enough for the job was present among a number of participants.

My biggest problem is, I think, applying for a job. And I need more time to adapt than other people, so for me it is hard to find a job and to keep the job…I am, you know, very unsure if I can find a job that suits my education. For sure, I can become a postman, but I think that would be a pity. (Woman, 32 years old).

### Perceived health during the COVID-19 crisis

In general, mean scores of SRH during the COVID-19 crisis were lower than mean scores before the COVID-19 crisis (Table 2). Mental SRH among those who lost their job decreased the most (by 14.3 percent). The biggest change score in financial distress was observed among those who lost their jobs, with a 18.8 percent change (increase in distress). The overall lowest change scores were observed among unemployed individuals. Among the unemployed, SRH (relatively low) and financial distress (highly present) did not change a lot compared to other groups. One-way ANOVA (Bonferroni posthoc tests) compared the mean scores during the COVID-19 crisis on SRH and financial distress in the four work status groups (see Table 2 annotations). Significant differences in mean scores during the COVID-19 crisis were observed in physical health (F = 25.55, *p* < 0.00), mental health (F = 14.81, *p* < 0.00) and financial distress (F = 7.85, *p* < 0.00). Bonferroni posthoc tests showed a significant difference in physical health between employed versus unemployed (*p* < 0.00) and unemployed versus became employed (*p* < 0.00). Similarly, mean mental health scores were significantly different between employed versus unemployed (*p* < 0.00) and unemployed versus became employed (*p* < 0.00). Regarding financial distress, significant differences were observed between employed versus unemployed (*p* = 0.05) and employed versus became unemployed (*p* = 0.04). Yet, all results should be interpreted carefully due to a small sample size among study participants that became employed or became unemployed.

**Table 2:** Self-rated health before and during the COVID-19 crisis among survey respondents (stratified by work status)

	Total (*N* = 584) Mean (SD)	Employed (*N* = 229) Mean (SD)	Unemployed (*N* = 263) Mean (SD)	Became unemployed (*N* = 35) Mean (SD)	Became employed (*N* = 57) Mean (SD)	One-way ANOVA
Self-rated physical health Range 1–5 (lowest to highest)						
Before COVID-19 crisis ^a b^	2.42 (0.97)	**2.78 (0.87)**	**2.04 (0.93)**	2.46 (0.95)	**2.71 (0.84)**	F = 29.26 (*p* < 0.00)
During COVID-19 crisis ^a b^	2.30 (0.95)	**2.63 (0.87)**	**1.95 (0.92)**	2.26 (0.85)	**2.58 (0.86)**	F = 25.55 (*p* < 0.00)
Difference in mean	0.12	0.15	0.09	0.20	0.13	
% change	- 5.0	-5.4	-4.4	-8.1	-4.8	
Self-rated mental health Range 1–5						
Before COVID-19 crisis ^a b c^	2.66 (0.97)	**3.00 (0.90)**	**2.32 (0.95)**	**2.80 (0.83)**	**2.77 (0.95)**	F = 22.94 (*p* < 0.00)
During COVID-19 crisis ^a b^	2.53 (0.95)	**2.78 (0.90)**	**2.26 (0.93)**	2.46 (0.92)	**2.77 (0.93)**	F = 14.81 (*p* < 0.00)
Difference in mean	0.13	0.18	0.06	0.40	0.00	
% change	-4.9	-6.0	-2.6	-14.3	0.0	
Self-rated financial distress						
Range 1–5						
Before COVID-19 crisis ^a^	2.71 (1.1)	**2.43 (0.91)**	**2.93 (1.11)**	2.60 (1.26)	2.82 (1.17)	F = 9.50 (*p* < 0.00)
During COVID-19 crisis ^a d^	2.81 (1.1)	**2.55 (0.95)**	**2.98 (1.14)**	**3.09 (1.29)**	2.96 (1.13)	F = 7.85 (*p* < 0.00)
Difference in mean	0.10	0.12	0.05	0.49	0.14	
% change	3.7	4.9	1.7	18.8	5.0	

^All statistical differences between comparison groups are presented in bold^

^a^Significant difference between unemployed vs. employed

^b^Significant difference between unemployed vs. became employed

^c^Significant difference between unemployed vs. became unemployed

^d^Significant difference between employed vs. became unemployed

### Individual experiences and perspectives related to health and social life

Although the COVID-19 crisis impacted all individuals differently, consequences on people’s health and their social life were mentioned by most participants. The thematic map of the sub-themes and main codes derived from the themes ‘Health’ and ‘Social life’ are presented in Additional file 2.

#### Mental distress and physical improvement

Qualitative findings revealed that participants experiences during the COVID-19 crisis had both negative and positive impacts on their health. While some participants mentioned more loneliness and/or depressive symptoms, others experienced more time to exercise due to loss of travel time to work.

…I pushed myself to exercise more, and I lost a lot of weight. So actually, my health is quite good now. I think COVID has been quite good for me personally. (Man, 28 years old)

People expect you will come to their birthday, that you will come by now and then. And then, suddenly, wow, you have a lot of time for yourself. I started to work on myself, to improve my health. Let’s see what interested me, how can I develop myself? (Man, 29 years old).

Additionally, a substantial portion of participants served as the caregiver for their partner or close relative. They felt compelled to limit social contact and only went out for necessities.

You were just afraid of infecting each other. And well, my father, he is very vulnerable. So I was actually forced to limit contact with other people, and only keep in contact with my clients [during work]. (Man, 29 years old).

Because she [my partner] is also chronically ill, we decided that I only go out to do groceries. (Man, 62 years old)

#### Social isolation

Complementary to the quantitative results, our qualitative findings showed that a feeling of social isolation increased among participants during the COVID-19 crisis. Individual interviews revealed that participants felt that they had to re-learn social skills after lockdown measures. Furthermore, contact with family or friends declined among a substantial part of participants. Study participants often mentioned the need to avoid crowded places.

…it’s been such a long time since I’ve spoken to someone. How do you do that, you know? Suddenly, it became very difficult to catch up, because life in a way changed so much. (Man, 28 years old)

Furthermore, conflicting relationships were frequent among a substantial part of participants. Friction in the work environment or conflicts with family members were often mentioned when participants were asked about receiving social support. Our findings show that these conflicting relationships were not only prevalent throughout the corona crisis but had already existed for a longer period.

### Relationship between work status and (changes in) self-rated health and financial distress

The relationship between (changes in) work status, SRH, and financial distress was analysed using logistic and linear regression (Table 3). Unemployed participants and participants who became unemployed during the COVID-19 crisis were less likely to perceive good physical and good mental health than employed participants. Table 3 shows the odds ratio (OR) for good self-rated health between subgroups (by work status) during the COVID-19 pandemic. Compared to employed participants, unemployed participants had substantially lower odds of perceiving themselves to have good physical health (OR = 0.29; 0.19, 0.43) and mental health (OR = 0.33; 0.22. 0.48). However, we found no significant relationship between becoming unemployed and physical or mental health. Compared to employed participants, participants in search of work or those who became employed were significantly more likely to perceive financial distress. These findings were finalized after adjusting for sex, age, educational level, and living situation. The association between work status and changes in self-rated health and financial distress were analysed with linear regression. In this analysis, the outcome variable was generated by using the change score of self-rated health and financial distress before and during the COVID-19 crisis. Those who had lost their job during the COVID-19 crisis were more likely to have a negative change in their perceived financial distress (% change = -0.36; -0.72, -0.01). Other change scores remained insignificant and relatively low among all subgroups of work status.

**Table 3:** Associations between work status, self-rated health, and financial distress

	Self-rated physical health		Self-rated mental health		Self-rated financial distress	
Work status	OR^a^	95% CI	OR^a^	95% CI	OR^b^	95% CI
Employed (Ref.)						
Became employed	0.90	0.48, 1.69	0.76	0.41, 1.39	**0.43**	**0.21, 0.87**
Became unemployed	0.52	0.24, 1.15	0.47	0.22, 1.00	**0.25**	**0.11, 0.56**
Unemployed	**0.29**	**0.19, 0.43**	**0.33**	**0.22, 0.48**	**0.30**	**0.19, 0.49**
	% change	95% CI	% change	95% CI	% change	95% CI
Employed (Ref.)						
Became employed	0.01	-0.16, 0.19	-0.20	-0.40, 0.01	-0.05	-0.33, 0.24
Became unemployed	0.09	-0.13, 0.30	0.19	-0.07, 0.44	**-0.36**	**-0.72, -0.01**
Unemployed	-0.05	-0.16, 0.06	-0.10	-0.23, 0.03	-0.06	-0.12, 0.24

^a^Response categories ‘excellent’, ‘very good’, and ‘good’ are merged into one ‘good’ category and ‘very poor’ and ‘poor’ are merged into one ‘poor’ category

^b^Response categories ‘very low’, ‘low’, and ‘neutral’ are merged into one ‘low’ category and ‘very high’ and ‘high’ are merged into one ‘high’ category. The values of *p* < 0.05 are highlighted in bold. Self-rated physical health, self-rated mental health, and self-rated financial distress were separately entered into the multivariate model. The model was adjusted for participants’ sex, age, educational level, and living situation (living alone/not alone)

## Discussion

The aim of this mixed methods study was to describe changes in work status, working conditions, and perceived health among persons with (partial) work disabilities in the Netherlands during the COVID-19 crisis and the associated restrictive and supportive measures. Survey results indicated that the vast majority of participants (84.2%) experienced no change in work status: 39.2 percent remained employed, 45.0 percent remained unemployed, six percent of participants lost their job during the COVID-19 crisis, and almost ten percent became employed. Negative changes in self-rated physical and mental health and financial distress were highest among those who lost their jobs. Although the majority of participants who received vocational rehabilitation (VR) were satisfied with the VR they received, those who remained unemployed were less often satisfied than those who remained employed. Findings derived from the linear regression showed weak associations between (changes in) work status and changes in self-rated health. The qualitative part of this study helped to gain more insight into individuals’ experiences and supported the quantitative differences in health outcomes between participants at work and in search of work. Interview findings also indicated that motivation to search for work decreased due to lack of structure and barriers in communication with potential employers during times of lockdown and restrictive measures.

The number of participants who lost their jobs remained low in this study. However, international studies found that unemployment, both among workers with and without disabilities, dramatically increased in the first months of the pandemic [8, 19]. Our relatively low observed numbers of job loss might be the result of the measures taken by the Dutch government to help businesses survive and protect employment at the time of the online survey (May and June 2021), including wage subsidies and loan guarantees [20]. Nevertheless, we found that employees were confronted with changed working conditions during the pandemic, such as reduced working hours. These findings are in line with results from the UK, where a reduction in working hours was observed and individuals with disabilities were found to be more likely to have their hours reduced during the COVID-19 crisis than their peers without disabilities [21]. Other employment consequences like not feeling safe at work, changes in work tasks, barriers in working from home, and difficulties communicating with colleagues and attending online meetings have also been emphasized by other studies of workers with disabilities and chronic health conditions [22, 23]. In our interviews with participants in search of work, structural barriers (such as self-stigma, social isolation, and conflicting relationships) were more frequently mentioned than COVID-19-related issues. This might be the result of structural and longer existing barriers, which were also described in other studies apart from the COVID-19 crisis [24–26] and which in turn became even more prominent in times of social distancing and reduced social support. However, these findings might also indicate that structural barriers are more impactful for this group of job seekers than the additional COVID-19-related problems.

In the present study it became clear that the COVID-19 crisis had both positive and negative consequences with regard to health. A number of health benefits were reported in the interviews with participants who were working from home, such as more time to exercise. However, social isolation and lack of contact with colleagues negatively affected work motivation and mental well-being. This is in line with studies providing evidence that social connectedness at work and support from co-workers and supervisors are important for mental health [27, 28]. Those in search of work seemed to be especially affected by social isolation and loneliness, which has likewise been demonstrated in other studies [29, 30]. In this study, barriers to participating in social life after a long period of social distancing were emphasized by unemployed individuals.

The main strength of our study is its mixed methods design. Our qualitative findings helped us to gain deeper understanding into the perspectives of individuals with work disabilities during the COVID-19 crisis and enriched our quantitative results. An additional strength was our cooperation with Expert by Experience co-researchers. The co-researchers were involved in designing and validating the survey and interview guidelines, and they also cooperated in carrying out the interviews. However, several limitations of this study must be acknowledged. An important limitation of this study is that all quantitative data was cross-sectional and self-reported, such that recall and information bias may have occurred. Secondly, it should be noted that the number of participants in this study is relatively low and that we did not take sectoral differences into account. Thirdly, the present study did not include the most hard-to-reach populations considering the sampling methods (which were mainly facility-based and respondent-driven). We acknowledge that undersampling of more isolated people is a main reported concern [31]. Consequently, selection bias may have caused underestimation of changes in self-rated health. Likewise with our qualitative findings, hindering factors in work and negative health outcomes among study participants are most likely only tip of the iceberg. A final limitation is that the present study does not compare people with and without work disabilities. It is plausible that the economic shock related to COVID-19 may have had a greater impact on people with disabilities than non-disabled people [32] because people with WD are more likely to be employed in the informal sector and often have work arrangements that bring fewer protections and entitlements compared to workers without disabilities [33].

### Recommendations for practice, policy, and future research

The finding that most participants remained employed during the COVID-19 crisis can be considered positive. However, participants at work nonetheless encountered challenges in their working environments. Difficulties emerged both from COVID-19-related health risks and from changes in work because of COVID-19 measures. Although most changes in work were identified to be temporary, returning to work for individuals with WD is generally accompanied by difficulties such as fear of returning to work, lack of motivation, and non-assertiveness [34]. The long-term effects of the COVID-19 crisis on people with work disabilities, including the impact of prolonged changes in working conditions, have yet to be unravelled. It is likely that some changes in working conditions will remain part of working life, such as working from home more frequently. Opportunities with regards to telework may also arise for people with work disabilities. In fact, telework for people with disabilities has been promoted since the 1990s [35]. Yet, working from home may require adjusted workplace accommodations and specific guidance from the workplace. Therefore, a deeper understanding of the personal and work-related factors needed for finding and maintaining work in ‘the new normal’ is necessary. Innovative thinking among all other stakeholders (i.e., professionals, policymakers and employers) may provide a broader view on increasing employment among people with WD during the COVID-19 crisis and beyond. Additionally, the low percentage of unemployed people with work disabilities that received vocational rehabilitation deserves attention, as these services could be beneficial for this group as well. Finally, understanding and targeting social interaction dilemmas for people with work disabilities in the context of work in vocational rehabilitation is important and should be further explored in future research.

## Conclusion

This study examined the relationship between the COVID-19 crisis and work status, working conditions, and health among persons with a (partial) work disability. Most likely due to governmental support, most participants (84.2%) experienced no change in work status. Nonetheless, people both at work and in search of work encountered barriers regarding maintaining or (re)gaining employment. People with a (partial) work disability who lost their job during the crisis seem to be most affected in terms of health. Employment and health protections could be strengthened for people with a work disability in order to build resilience in times of crisis.

## Supplementary Information

**Additional file 1.  **Main theme ‘Work status’, including themes, sub-themes, and main codes.

**Additional file 2.  **‘Health’ and ‘Social life’including sub-themes and main codes.

## Data Availability

The datasets generated and analysed during the current study are not publicly available due privacy regulations but are available from the corresponding author on reasonable request.

## References

[1] Burgard SA, Lin KY. Bad Jobs, Bad Health? How Work and Working Conditions Contribute to Health Disparities. Am Behav Sci. 2013;57(8):2–12. 10.1177/0002764213487347 PMC3813007 24187340

[2] Benach J (2014). Precarious employment: understanding an emerging social determinant of health. Annu Rev Public Health.

[3] Gold PB, Macias C, Rodican CF (2016). Does competitive work improve quality of life for adults with severe mental illness? Evidence from a randomized trial of supported employment. J Behav Health Serv Res.

[4] Perkins DV (2009). Gainful employment reduces stigma toward people recovering from schizophrenia. Community Ment Health J.

[5] Burns T (2009). The impact of supported employment and working on clinical and social functioning: results of an international study of individual placement and support. Schizophr Bull.

[6] Prins R, Loisel P, Anema J (2013). Sickness Absence and Disability: An International Perspective. Handbook of Work Disability.

[7] Putter I de, Cozijnsen R, Rijken M. Het vergroten van arbeidsparticipatie onder mensen met een chronische ziekten of beperking: een werkwens alleen is niet voldoende. www.nivel.nl: NIVEL. 2015.

[8] KsinanJiskrova G (2021). Job loss and lower healthcare utilisation due to COVID-19 among older adults across 27 European countries. J Epidemiol Community Health.

[9] Dang HH, Viet Nguyen C (2021). Gender inequality during the COVID-19 pandemic: Income, expenditure, savings, and job loss. World Dev.

[10] Ojala S, Pyöriä P (2019). Precarious work and the risk of receiving a disability pension. Scand J Public Health.

[11] Kouwenhoven-Pasmooij TA (2016). Cardiovascular disease, diabetes and early exit from paid employment in Europe; the impact of work-related factors. Int J Cardiol.

[12] van der Zwan R, de Beer P. The disability employment gap in European countries: What is the role of labour market policy? J Eur Public Policy. 2021;31(4):473–86.

[13] Dalise S, Tramonti F, Armienti E, Niccolini V, Caniglia-Tenaglia M, Morganti R, Chisari C. Psycho-social impact of social distancing and isolation due to the COVID-19 containment measures on patients with physical disabilities. Eur J Phys Rehabil Med. 2021;57(1):158–65. 10.23736/S1973-9087.20.06535-1. 10.23736/S1973-9087.20.06535-1 33165314

[14] Giorgi G (2020). COVID-19-Related Mental Health Effects in the Workplace: a narrative review. Int J Environ Res Public Health.

[15] Guerin RJ (2021). Investigating the Impact of Job Loss and Decreased Work Hours on Physical and Mental Health Outcomes Among US Adults During the COVID-19 Pandemic. J Occup Environ Med.

[16] Griffiths D (2021). The Impact of Work Loss on Mental and Physical Health During the COVID-19 Pandemic: Baseline Findings from a Prospective Cohort Study. J Occup Rehabil.

[17] Mortazavi SS (2020). Fear, Loss, Social Isolation, and Incomplete Grief Due to COVID-19: A Recipe for a Psychiatric Pandemic. Basic Clin Neurosci.

[18] van der Velden PG (2020). Anxiety and depression symptoms, and lack of emotional support among the general population before and during the COVID-19 pandemic. A prospective national study on prevalence and risk factors. J Affect Disord.

[19] Houtenville AJ, Paul S, Brucker DL (2021). Changes in the employment status of people with and without disabilities in the United States During the COVID-19 Pandemic. Arch Phys Med Rehabil.

[20] CPB COVID-19 Publication – Business Dynamics During The COVID Pandemic. www.cpb.nl: Sociaal Cultureel Planbureau, april 2021.

[21] Emerson E (2021). The impact of disability on employment and financial security following the outbreak of the 2020 COVID-19 pandemic in the UK. J Public Health (Oxf).

[22] Wong J, et al. Employment Consequences of COVID-19 for People with Disabilities and Employers. J Occup Rehabil. 2022;32(3):464–72. 10.1007/s10926-021-10012-9 PMC8761523 35037157

[23] Schwartz AE (2021). Impact of COVID-19 on services for people with disabilities and chronic health conditions. Disabil Health J.

[24] Lettieri A (2022). Employment related barriers and facilitators for people with psychiatric disabilities in Spain. Work.

[25] Small SP, de Boer C, Swab M (2022). Barriers to and facilitators of labor market engagement for individuals with chronic physical illnesses in their experiences with work disability policy: a qualitative systematic review. JBI Evidence Synthesis.

[26] Maddineshat M (2022). Facilitators and Barriers of Return to Work in Working People with Serious Mental Illness: A Qualitative Study. Indian J Psychol Med.

[27] Shahidi FV (2021). Assessing the psychosocial work environment in relation to mental health: a comprehensive approach. Ann Work Expo Health.

[28] Finne LB, Christensen JO, Knardahl S (2014). Psychological and social work factors as predictors of mental distress: a prospective study. PLoS One.

[29] Bell LM (2021). COVID-19 stressors, wellbeing and health behaviours: a cross-sectional study. J Public Health.

[30] Lee JO (2021). Estimating influences of unemployment and underemployment on mental health during the COVID-19 pandemic: who suffers the most?. Public Health.

[31] Shaghaghi A, Bhopal RS, Sheikh A. Approaches to recruiting “hard-to-reach” populations into re-search: a review of the literature. Health Promot Perspect. 2011;1(2):86–94. 10.5681/hpp.2011.009 PMC3963617 24688904

[32] Gignac MAM (2021). Impacts of the COVID-19 pandemic on health, financial worries, and perceived organizational support among people living with disabilities in Canada. Disabil Health J.

[33] Maroto M, Pettinicchio D (2014). Disability, structural inequality, and work: The influence of occupational segregation on earnings for people with different disabilities. Res Soc Stratif Mobil.

[34] Joosen MCW, et al. Barriers and facilitators for return to work from the perspective of workers with common mental disorders with short, medium and long-term sickness absence: a longitudinal qualitative study. J Occup Rehabil. 2022;32(2):272–83. 10.1007/s10926-021-10004-9 PMC9232415 34580811

[35] Moon NW (2014). Telework rationale and implementation for people with disabilities: considerations for employer policymaking. Work.

