# Supplementary material for: Work and health during the COVID-19 crisis among Dutch workers and jobseekers with (partial) work disabilities: a mixed methods study
Source: BMC Public Health. 2023 May 26;23:966. doi: 10.1186/s12889-023-15720-w (PMC10214321; doi:10.1186/s12889-023-15720-w)
Supplement: Supplementary file 1 — Additional file 1. Main theme ‘Work status’, including themes, sub-themes, and main codes. [file 12889_2023_15720_MOESM1_ESM.docx]

Additional file 1: Main theme ‘Work status’, including themes, sub-themes, and main codes

Main theme Themes Sub-themes Main codes

Additional figure 1 | Main theme ‘Work status’, including themes, sub-themes and main codes
