# Supplementary material for: Work and health during the COVID-19 crisis among Dutch workers and jobseekers with (partial) work disabilities: a mixed methods study
Source: BMC Public Health. 2023 May 26;23:966. doi: 10.1186/s12889-023-15720-w (PMC10214321; doi:10.1186/s12889-023-15720-w)
Supplement: Supplementary file 2 — Additional file 2. ‘Health’ and ‘Social life’including sub-themes and main codes. [file 12889_2023_15720_MOESM2_ESM.docx]

Additional file 2: **‘**Health’ and ‘Social life’ including sub-themes and main codes

Themes Sub-themes Main codes

Additional figure 2| ‘Health’ and ‘Social life’ including sub-themes and main codes
